# Updated Oxford classification and the international study of kidney disease in children classification: application in predicting outcome of Henoch-Schönlein purpura nephritis

**DOI:** 10.1186/s13000-019-0818-0

**Published:** 2019-05-10

**Authors:** Xiaohan Huang, Lili Ma, Pingping Ren, Hongya Wang, Liangliang Chen, Haidongqin Han, Jianghua Chen, Fei Han

**Affiliations:** 1 Kidney Disease Center, The First Affiliated Hospital, College of Medicine, Zhejiang University, 79 Qingchun Road, Hangzhou, Zhejiang Province 310003 People’s Republic of China; 2 Institute of Nephrology, Zhejiang University, Hangzhou, Zhejiang Province China; 3 Key Laboratory of Kidney Disease Prevention and Control Technology, Hangzhou, Zhejiang Province China; 4 The Third Grade Laboratory under the National State Administration of Traditional Chinese Medicine, Hangzhou, China; 5 Department of Nephrology, Shaoxing Traditional Chinese Medicine Hospital, Shaoxing, China

**Keywords:** IgA vasculitis, Nephritis, Renal biopsy, Crescent, Outcomes

## Abstract

**Background:**

Henoch-Schönlein purpura nephritis (HSPN) shares many similarities with IgA nephropathy. We aimed to analyze the predictive value of the International Study of Kidney Disease in Children (ISKDC) classification and the updated Oxford classification for IgA nephropathy in HSPN patients.

**Methods:**

Data of 275 HSPN patients (aged≥14 years) were retrieved, and all of them underwent a renal biopsy. We re-classified the biopsies according to the ISKDC classification and the updated Oxford classification to analyze their correlations with clinical features and renal outcomes. The renal endpoints were defined as ≥30% reduction in baseline estimated glomerular filtration rate (eGFR) in 2 years, doubling of serum creatinine (Scr) or end stage renal disease.

**Results:**

During follow-up period of 56(30,86) months, 30(10.9%) patients reached renal endpoints. Segmental sclerosis was the only pathological feature independently associated with renal endpoints (HR 4.086, 95%CI 1.111–15.026, *P* = 0.034). Tubular atrophy/ interstitial fibrosis was associated with eGFR and Scr levels, and its correlation with renal endpoints was found by univariate analysis. Endocapillary hypercellularity was associated with 24 h urine protein and is of prognostic value in univariate analysis. Mesangial hypercellularity was not associated with clinical features or renal endpoints. Crescents were associated with 24 h urine protein, Scr and eGFR levels, but both ISKDC and updated Oxford classifications of crescents were not associated with renal endpoints by multivariate analysis.

**Conclusions:**

The updated Oxford classification can help in disease management and renal outcome prediction of HSPN.

**Electronic supplementary material:**

The online version of this article (10.1186/s13000-019-0818-0) contains supplementary material, which is available to authorized users.

## Background

Henoch-Schönlein purpura (HSP), which also known as IgA vasculitis (IgAV), is an immunoglobulin A (IgA)-mediated disease characterized by a generalized vasculitis mainly involving the skin, joints, gastrointestinal tract and kidneys [1]. IgAV nephritis (IgAVN) or Henoch-Schönlein purpura nephritis (HSPN) is the kidney damage caused by IgAV. It has been reported that 7–23% of children and 10–27% of adults with HSPN would progress to end stage renal disease (ESRD) [2–5]. For HSP patients, a major factor affecting the long-term outcome is the severity of renal involvement [6], and it has been suggested that some pathologic features may have values in predicting the outcomes of HSPN [7].

Pathologic features of HSPN is usually graded by the International Study of Kidney Disease in Children (ISKDC) classification, mainly according to the existence and the number of crescents [8]. However, the value of crescents in predicting long-term outcome of HSPN is still lack of consensus. IgA nephropathy shares many similarities with HSPN in clinical, immunological and histological features [9]. In some studies, IgA nephropathy and HSPN were regarded as different manifestations of a single disease [10, 11]. Therefore, the Oxford classification of IgA nephropathy may also be used to group pathologic features of HSPN. The updated Oxford classification uses mesangial hypercellularity (M), endocapillary hypercellularity (E), segmental sclerosis (S), tubular atrophy/ interstitial fibrosis (T) and crescents (C) to evaluate renal biopsies [12]. A study published in 2014 suggested that the original Oxford classification was of prognostic value in HSPN [13],while another study in 2018 suggested that updated oxford classification can be used to assess renal outcomes of HSPN in children [14]. Here, we evaluated the utility of both the ISKDC classification and the updated Oxford classification as a predictor of renal outcome by retrospectively reviewing a larger cohort (275 patients, aged≥14 years)followed up in our center.

## Methods

### Patients

Data of 292 HSPN patients diagnosed by the Kidney Disease Center of the First Affiliated Hospital of Zhejiang University between August 2004 and November 2015 were retrieved. Criteria for a renal biopsy was HSPN patients who had active urinary sediments (proteinuria or hematuria) with or without impairment of glomerular filtration rate. Inclusive criteria included: aged ≥14 years; a history of palpable purpuric eruption, with or without involvement of gastrointestinal tract or joints; biopsy-proven HSPN. Exclusion criteria was as follows: inadequate number of glomeruli (< 8) in biopsy, concomitant with cirrhotic liver disease, IgA-dominant infection associated glomerulonephritis, human immunodeficiency virus associated IgA nephropathy, anti-neutrophil cytoplasmic antibody associated vasculitis, hepatitis B virus associated nephritis, idiopathic thrombocytopenic purpura, systemic lupus erythematosus and other autoimmune diseases. Levels of antinuclear antibody (ANA), anti-neutrophil cytoplasmic antibody (ANCA), anti-glomerular basement antibody together with complements were collected to exclude autoimmune disease.

Of these 292 patients, 11 patients were lost to follow-up, and another 6 patients were excluded because of biopsy inadequacy (< 8 glomeruli), the remaining 275 patients were the basis of our study. The study protocols conformed to the provisions of the Declaration of Helsinki. The Research Ethics Committee of the First Affiliated Hospital, College of Medicine, Zhejiang University approved the protocols (Reference number: 2017678).

### Clinical data collection

Medical records of the patients were reviewed to obtain clinical data at the time of biopsy and during the course of follow-up. The data included age, gender, blood pressure, urine protein excretion based on 24-h urine collection, serum creatinine (Scr) and estimated glomerular filtration rate (eGFR). Also, the use of immunosuppressant drugs was assessed. The mean arterial pressure (MAP) was calculated by the equation MAP = (2 × diastolic pressure + systolic pressure)/3, while the eGFR was calculated using the Chronic Kidney Disease Epidemiology Collaboration (CKD-EPI) formula.

### Pathologic data collection

Renal biopsy of all patients were graded according to the International Study of Kidney Disease in Children classification [8]: (I) minimal glomerular alterations; (II) mesangial proliferation; (IIIa) focal or (IIIb) diffuse proliferation or sclerosis with < 50% crescents; (IV) mesangial proliferation or sclerosis with 50–75% crescents; (V) mesangial proliferation or sclerosis with > 75% crescents; (VI) membranoproliferative-like lesion. Patients were divided into three groups: Group ISKDC-G0 included ISKDC grade I and II, Group ISKDC-G1 included ISKDC grade III, and ISKDC-G2 included ISKDC grade IV, V and VI. Pathologic findings in these renal biopsies were then re-evaluated with the updated Oxford classification MEST-C scoring system [12]:(1) mesangial proliferation (M): mesangial score < 0.5 (M0), or > 0.5(M1); (2) endocapillary hypercellularity (E): absent (E0) or present (E1); (3) segmental glomerulosclerosis (S): absent (S0) or present (S1); (4) tubular atrophy/ interstitial fibrosis (T): 0–25% (T0), 25–50% (T1), > 50% (T2); (5)cellular/ fibrocellular crescents (C): absent (C0), 0–25% (C1), ≥25% (C2). Group T1 and T2 were merged because there were only 2 patients scored T2. One experienced renal pathologist who was blinded to the case histories evaluated all renal pathology.

### Definitions

The primary renal endpoint was defined as ≥30% reduction of eGFR from baseline in 2 years, doubling of Scr or ESRD. ESRD was defined by reaching an eGFR of <15 ml/min/1.72m^2^ or requiring maintaining renal replacement therapy for more than 3 months.

### Statistical analysis

Statistical analysis was performed by IBM SPSS 22.0 (IBM, Chicago, IL, USA). Normal distributed data were expressed as mean ± standard deviation, and comparisons were tested by Student’s t test. Non-normal distributed data were expressed as median(interquartile range), and compared by the Mann-Whitney U-test. Chi-square *χ*^2^ test, Fisher’s exact test and non-parametric test were used to compare categorical variables. We used one-way ANOVA and R x C contingency table for comparison of three groups. Student-Newman-Keuls test was used to further compare between two groups. To evaluate the association between the classifications and renal endpoint, we used univariate analysis and multivariate Cox regression model, defining the day of renal biopsy as starting point, and the result was presented with hazard ratios (HR). *P* < 0.05 was considered significant.

## Results

### Demographic and clinical features

The study sample comprised 275 HSPN patients. At the time of biopsy, their mean age was 33 ± 17 years, and 52.0% were male. All of the patients were the Han population. During a median follow-up of 56(30,86) months, 30(10.9%) patients reached renal endpoints including more than 30% decrease in baseline eGFR in 2 years (11 patients), doubling in Scr (7 patients) and ESRD (12 patients). Comparations between patients with or without renal endpoints were shown in Table 1. There were no significant differences in age and extrarenal manifestations, but patients reaching endpoints had a higher male proportion, higher average MAP (100 ± 9 mmHg vs 94 ± 11 mmHg, *p* = 0.012), higher Scr level [103.0 (74.8217.8) μmol/L vs 63.0 (53.0,78.0) μmol/L, *p* < 0.001] and lower eGFR level [67.4 (30.1, 115.4) ml/min/1.73m^2^ vs 121.2 (92.6, 140.1)ml/min/1.73m^2^, *p* < 0.001], and more proteinuria [2.7 (1.0, 5.0) g/24 h vs 1.12 (0.6, 2.7) g/24 h, *p* < 0.001]. Angiotensin converting enzyme inhibitor (ACEI) / angiotensin II receptor blocker (ARB) and immunosuppressants were prescribed in our patients. A percentage of 72.7% of patients received immunosuppressive therapy; the percentage was same in patients with or without renal endpoints. However, patients with endpoints received a higher proportion of steroids combining with intravenous cyclophosphamide or mycophenolate mofetil (18/24 vs 83/176, *p* = 0.019), probably correlating with a higher severity of the disease.

**Table 1:** Clinical features for patients with or without renal endpoints

Clinical features	Renal endpoints reached		*P* value
	Yes	No	
Number of patients	30	245	
Mean age (years)	42 ± 22	33 ± 16	0.052
Gender (Male/Female)	21/9	122/123	0.036
Extrarenal manifestation			
Skin (n, %)	30 (100%)	245 (100%)	
Gastrointestinal tract (n, %)	4 (13.3%)	62 (24.3%)	0.147
Joints (n, %)	1 (3.3%)	45 (18.4%)	0.068
MAP (mmHg)	100 ± 9	94 ± 11	0.012
Scr (μmol/L)	103.0 (74.8217.8)	63.0 (53.0,78.0)	<0.001
eGFR (ml/min/1.73m^2^)	67.4 (30.1, 115.4)	121.2 (92.6, 140.1)	<0.001
Proteinuria(g/24 h)	2.7 (1.0, 5.0)	1.12 (0.6, 2.7)	<0.001
Immunosuppressant therapy (n, %)	24 (80.0%)	176 (71.8%)	0.343
Steroids only(n)	6	93	0.019
Steroids + IVC/MMF(n)	18	83	
ACEI/ARB	12 (40.0%)	83 (33.9%)	0.51

The data were expressed as mean ± s.d. or as numbers(percentage)

Results of Scr, eGFR, proteinuria were expressed as median (interquartile range)

*MAP* mean arterial pressure, *Scr* serum creatinine, *eGFR* estimated glomerular filtration rate, *IVC* intravenous cyclophosphamide, *MMF* mycophenolate mofetil, *ACEI* angiotensin converting enzyme inhibitor, *ARB* angiotensin II receptor blocker

The detailed pathologic features (including the severity of mesangial proliferation, the proportion of glomeruli with each pathologic lesion, and the proportion of interstitial inflammation) in patients with or without renal endpoints were shown in Table 2. A greater proportion of glomeruli with crescents (*p* < 0.001), and higher percentages of tubular atrophy (*p* = 0.013) and tubulointerstitial inflammation (*P* = 0.004) were observed in patients reaching renal endpoints. Immunofluorescence deposition feature is provided in Additiobnal file 1: Table S1

**Table 2:** Detailed pathologic features for patients with or without renal endpoints

Pathologic features	Renal endpoints reached		*P* value
	Yes	No	
Mesangial hypercellularity ^a^, n (%)			0.238
Normal	0	2 (0.4)	
Mild	26 (86.7)	207 (84.5)	
Moderate	3 (10.0)	35 (14.3)	
Severe	1 (3.3)	1 (0.4)	
tubulointerstitial inflammation ^a,b^, n (%)			0.004
Absent	4 (13.3)	68 (27.7)	
≤25%	12 (40.0)	133 (54.3)	
>25% and ≤ 50%	6 (20.0)	12 (4.9)	
>50%	8 (26.7)	32 (13.1)	
Proportion of glomeruli per biopsy			
Segmental sclerosis (%)^c^	3.4 (0, 16.7)	2.0 (0, 7.5)	0.098
Crescents (%)^c^	12.9 (3.5, 23.3)	4.0 (0, 13.3)	<0.001
Tubular atrophy (%)^c^	5 (1, 15)	5 (0, 5)	0.013

The data was expressed as numbers (percentages)

^a^Mesangial hypercellularity was assessed in semi- quantitative way: normal = 3 or less cells per mesangial area; mild = 4 or 5 cells per mesangial area; moderate = 6 or 7 cells per mesangial area; severe = 8 or more cells in mesangial area

^b^Tubulointerstitial inflammation applied to inflammation in the biopsy overall (including areas of interstitial fibrosis/tubular atrophy)

^c^Proportion of glomeruli per biopsy with lesion, data was expressed as median (interquartile range)

Distribution of the patients categorized by the ISKDC classification and the updated Oxford classification was presented in Table 3. There were 101(36.7%) patients graded ISKDC II, 148(53.8%) patients graded IIIa, 17(6.2%) patients graded IIIb, 8(2.9%) patients graded IV, and only 1(0.4%) patients graded V. No patient was graded I or VI. By the updated Oxford classification, M1, E1, S1, T1/T2 were observed in 41(14.9%), 82(29.8%), 149(54.2%), 8(2.9%) patients respectively. Additionally, there were only 6 patients graded T1, and 2 patients graded T2, so we merged them in a group (T1/T2) for analysis. With regards to crescents, 141(51.3%) patients scored C1 and 35(12.7%) patients scored C2. For renal outcomes, the percentage of patients reaching renal endpoints increased with the increasing grade from ISKDC-G0, G1 to G2 (4.9, 12.7, 44.4% respectively, *p* < 0.001), and those in M1, E1, S1, T1/T2, C1 or C2 groups of the updated Oxford classification were 12.1, 15.8, 12.7, 25, 13.4% or 20%, respectively.

**Table 3:** Distribution of patients classified according to the updated Oxford classification or the International Study of Kidney Disease in Children classification

Classification	All patients^a^	Patients reaching endpoints^b^	*P* value
*International Study of Kidney Disease in Children classification*			
I	0	0	
II	101 (36.7%)	5 (4.9%)	
IIIa	148 (53.8%)	18 (12.2%)	
IIIb	17 (6.2%)	3 (17.6%)	
IV	8 (2.9%)	3 (37.5%)	
V	1 (0.4%)	1 (100%)	
VI	0	0	
ISKDC-G0	101 (36.7%)	5 (4.9%)	<0.001
ISKDC-G1	165 (60.0%)	21 (12.7%)	
ISKDC-G2	9 (3.3%)	4 (44.4%)	
*Updated Oxford classification*			
M0	234 (85.1%)	25 (10.6%)	0.775
M1	41 (14.9%)	5 (12.1%)	
E0	193 (70.2%)	17 (8.8%)	0.086
E1	82 (29.8%)	13 (15.8%)	
S0	126 (45.8%)	11 (8.7%)	0.287
S1	149 (54.2%)	19 (12.7%)	
T0	267 (97.1%)	28 (10.4%)	0.194
T1/T2	8 (2.9%)	2 (25.0%)	
C0	99 (36.0%)	4 (4.0%)	0.011^c^
C1	141 (51.3%)	19 (13.4%)	0.386^d^
C2	35 (12.7%)	7 (20.0%)	

Data was expressed as numbers(percentages)

^a^Number of patients in the group, and the proportion in all patients

^b^Number of patients reached endpoint and the proportion in this group

^c^C0 compares to C1 + C2, crescent absent vs crescent formation

^d^C0, C1, C2, comparison made by contingency table

### Correlations between clinical data and pathologic classifications

Correlations between clinical data and pathologic classifications were shown in Table 4. According to the ISKDC classification, with the increase of classification from ISKDC G0, G1 to G2, patients had more daily urine protein (*p* < 0.05), higher Scr level (*p* < 0.05) and lower eGFR level (*p* < 0.05). According to the updated Oxford classification, no significant difference in MAP, proteinuria, eGFR and Scr levels was found between M0 and M1 group; patients in E1 group had more daily urine protein than E0 group (*p* < 0.001); patients in S1 group had higher MAP level (*p* = 0.003), higher Scr level (*p* < 0.001) and lower eGFR level (p < 0.001) than those in S0 group; patients in T1/T2 group had higher Scr level (*p* = 0.001) and lower eGFR level (p = 0.001) than those in T0 group; both patients in C1 group (Crescents 0–25%) and C2 group (Crescents ≥25%) had more daily urine protein (*p* < 0.05), higher Scr level (*p* < 0.05) and lower eGFR level (*p* < 0.05) than those in C0 group; furthermore, patients in C2 group have more proteinuria than C1 group.

**Table 4:** Correlations between clinical data and pathologic classifications by the updated Oxford classification or the International Study of Kidney Disease in Children classification

	MAP, mmHg	P value	Proteinuria, g/24 h	*P* value	eGFR, ml/min/1.73m^2^	*P* value	Scr, μmol/L	P value
*International Study of Kidney Disease in Children classification*								
ISKDC-G0	93.8 ± 10.7		0.75 (0.40, 1.75)		123.8 (96.0, 145.4)		60.0 (50.5, 75.0)	
ISKDC-G1	95.9 ± 10.6		1.50 (0.75, 3.00) ^a^		111.4 (81.4, 134.6) ^a^		68.0 (55.0, 91.5) ^a^	
ISKDC-G2	95.0 ± 10.5		3.60 (2.00, 5.50)^ab^		30.7 (24.5, 138.6)^ab^		150.0 (56.0, 244.5)^ab^	
*Updated Oxford classification*								
Messangial proliferation								
M0	95.1 ± 10.4	0.303	1.23 (0.60, 3.00)	0.859	116.2 (87.6, 139.6)	0.429	65.0 (53.0, 81.3)	0.098
M1	94.3 ± 12.4		1.34 (0.73, 3.01)		107.3 (85.5, 134.7)		72.0 (56.0, 92.0)	
Endocapillary proliferation								
E0	94.5 ± 10.3	0.160	1.00 (0.46, 2.15)	< 0.001	118.6 (89.7, 136.3)	0.798	65.0 (54.0, 84.0)	0.946
E1	96.2 ± 11.4		2.02 (1.00, 3.44)		110.4 (80.5, 145.3)		66.0 (52.0, 84.3)	
Segmental sclerosis								
S0	91.5 ± 10.2	0.003	1.06 (0.52, 3.00)	0.423	130.4 (109.3, 144.9)	< 0.001	60.3 (51.0, 75.0)	< 0.001
S1	96.8 ± 10.8		1.50 (0.70, 3.00)		99.0 (75.2, 125.1)		68.5 (59.3, 94.0)	
Tubular atrophy/ interstitial fibrosis								
T0	94.9 ± 10.7	0.148	1.30 (0.64, 3.00)	0.273	118.9 (89.8, 139.4)	0.001	65.0 (54.0, 81.0)	0.001
T1/T2	99.4 ± 7.1		2.72 (1.00, 3.43)		45.3 (18.4, 76.7)		138.5 (106.8, 276.0)	
Crescents								
C0	93.6 ± 10.7		0.75 (0.42, 1.97)		124.2 (96. 5, 145.6)		59.1 (50.7, 74.0)	
C1	95.9 ± 11.0		1.50 (0.75, 3.05)^c^		114.8 (81.9, 134.8)^c^		70.2 (54.0, 89.0)^c^	
C2	95.1 ± 8.8		2.49 (1.33, 4.65)^cd^		95.9 (41.9, 131.9)^c^		68.6 (57.0, 150.5)^c^	

MAP, mean arterial pressure; Scr, serum creatinine; eGFR, estimated glomerular filtration rate; ISKDC, International Study of Kidney Disease in Children. ^a^ *P* < 0.05 compared with ISKDC-G0, ^b^ *P* < 0.05 compared with ISKDC-G1; ^c^ *P* < 0.05compared with C0, ^d^ *P* < 0.05 compared with C1

Results of MAP were provided in mean ± standard deviation while results of Scr, eGFR, proteinuria were provided in median and (interquartile range)

### Correlations between outcomes and pathologic classifications

Survival analysis was shown in Table 5. By the univariate Cox regression analysis, E1(*P* = 0.038), S1(*p* = 0.005), T1/T2(p < 0.001) and C1/C2(p = 0.005) were risk factors for renal endpoints. In multivariate model, variants including age, 24-h proteinuria, eGFR, and pathologic features (E1, S1, T1/T2 and C1/C2) were analyzed; and S1 lesion (HR = 4.086 95%CI = 1.111–15.026, *p* = 0.034) and more proteinuria (HR = 1.191, 95%CI = 1.012–1.401, *p* = 0.035) were independently risk factors for renal endpoints.

**Table 5:** Cox model analysis for renal endpoints

Factors	univariate			Multivariate^a^		
	HR	95%CI	*P* value	HR	95%CI	*P* value
ISKDC G1/G2	2.359	0.868–6.411	0.092	–	–	–
M1	0.979	0.326–3.873	0.854	–	–	–
E1	2.447	1.051–5.697	0.038	1.527	0.571–4.082	0.399
S1	3.597	1.196–10.717	0.023	4.086	1.111–15.026	0.034
T1/T2	9.834	2.749–35.173	<0.001	2.605	0.546–12.434	0.230
C1/C2	2.387	1.296–4.394	0.005	1.757	0.858–3.598	0.123
Age	1.030	1.007–1.054	0.010	1.007	0.981–1.034	0.590
Proteinuria	1.196	1.059–1.351	0.004	1.191	1.012–1.401	0.035
eGFR	0.979	0.969–0.991	<0.001	0.993	0.980–1.006	0.284

^a^The model adjusted for age, Proteinuria, eGFR

## Discussion

We showed that the S lesion was an independent risk factor for poor renal outcome in HSPN. This was in agreement with previous studies in HSPN [3, 14] and IgA nephropathy including the oxford cohort [15–18]. The S lesion is considered to represent chronic and late stage kidney damage; therefore, the presence of S lesion can be valuable in predicting renal outcome of HSPN.

Tubular and interstitial lesions were not mentioned in ISKDC classification but were regarded as independent risk factors of IgA nephropathy according to the Oxford classification [12], validated by following studies [18–20]. The T lesions were related to higher Scr level and lower eGFR level, but not proven to be an independent risk factor of poor renal outcome in the present study. The degree of tubulointerstitial inflammation was associated with poor outcome, and by univariate Cox analysis, tubular atrophy and interstitial fibrosis were associated with poor renal prognosis, but Cox multivariate regression analysis failed to demonstrate the correlation. This result agreed with the study of Inagaki et al [21]. However, we must mention that the inadequacy of sample (only 8 patients were categorized in T1/T2 group) may mask the prognostic value of the T lesions. In the study of Kim et al [13] tubular atrophy and interstitial fibrosis were suggested to be an independent risk factor, but the number of cases in their study was also limited (8 patients). This may indicate that T1 or T2 lesion were relatively rare in HSPN patients.

In our study, mesangial proliferation was not related with renal outcome. This is consistent with previous studies, including a new classification for predicting outcomes of HSPN raised by Mikael K et al [22]. Thus, although the M lesion was confirmed to be a significant predicting factor in IgA nephropathy, its value in HSPN is still lack of evidence.

Patients presented with endocapillary hypercellularity had larger amount of 24 h urine protein excretion. The degree of proteinuria is proved to be a significant prognostic factor in our study and studies reported [3, 8, 23]. And, the E lesion was found of prognostic value in univariate cox model in our study. Glomerular endothelial cells are important components of the renal filtration barrier and the E lesion can damage the filtration barrier, letting protein leak into the urine, thus increase urine protein excretion. At the same time, the E lesion is considered to be an acute lesion related with inflammation, which can be reversed by immunosuppressant drugs or steroid therapy, so its chronic effect on GFR may be limited [24], and its correlation with renal outcome is still unclear. Our study, which found the E lesion was not independently associated with outcome, is consistent with the oxford classification study [16] and the VALIGA cohort [25] of IgA nephropathy. But it is in the contrary to studies with smaller patient population from Korea and Japan [13, 21]. Some studies had suggested that immunosuppressive therapy might affect the prognostic value of the E lesion in IgA nephropathy [26–28], so the high proportion of immunosuppressive therapy in our study may improve the effect of E lesion on renal outcome.

Crescents were also associated with more daily urine protein excretion, higher Scr and lower eGFR, supporting that the number of crescents was related with severity of clinical manifestation of HSPN [2, 29, 30]. The utility of crescents as a predictor of renal outcome in HSPN was long-debated. The formation of crescents has been a main grading parameter in the ISKDC classification since 1977; however, its correlation with renal outcome was not clear. Crescents were related with poor prognosis in some studies [2, 30, 31], but it was not confirmed by others [3, 13, 23]. Similar situation occurs in IgA nephropathy [32–34]. Crescents were relatively rare in IgA patients and were not used as a parameter in the Oxford classification until its recent update. The researchers also pointed out that only in those who did not receive immunosuppression was the association between crescents and poor prognosis statistically significant [12]. In our study, crescents graded by both the ISKDC and the Oxford classification was not independently related with renal outcome, probably correlating with the high proportion of immunosuppressive therapy (72.7%).

Our study shared some similarities with those done by Kim et al [13] and Xu et al [14]. In Kim’s study, E lesion and T lesions were suggested to be of prognostic value, while in Xu’s study, S and T lesions were of clinical value in outcome prediction. Possible factors which may lead to the difference were mentioned in previous paragraphs. Additionally, the disparity of patient population, follow-up duration and classification may also lead to the difference. Our study included a larger cohort (273 patients vs 92 patients or 104 patients respectively) and had longer median follow-up duration (56.0 months vs 49.3 months or 40 months respectively). Patients in our study were 14 years old or older (characteristics and survival curve of different age groups were provieded in Additional file 2: Table S2 and Additional file 3: Figure S1), while in Kim’s study the inclusive criteria was aged ≥16 years, and Xu’s study focused on pediatric patients. Also, we used updated Oxford classification instead of the original one, and evaluated the value of ISKDC classification as well.

There were several limitations in our study. Despite many similarities shared by HSPN and IgA nephropathy, there are still differences existing between them. Diffused endocapillary hypercellularity and crescents for example, are observed more common in HSPN than IgA nephropathy [9]. Besides, as a single-center retrospective study, selection bias was unavoidable. Also, the median follow-up duration of was 56 months, which was relatively shorter to observe more endpoint events. And the inadequacy of patient number, especially patients with T1 or T2 lesions, may restrict the accuracy of our study.

## Conclusion

The Oxford classification, especially the S score was helpful to evaluate the renal outcome of HSPN and can be used in disease management together with ISKDC classification.

## Additional files

Additional file 1: **Table S1.** Clinical and pathological features of patients in different age group. (DOCX 17 kb)

Additional file 2: **Table S2.** Immunofluorescence deposition characteristics. (DOCX 15 kb)

Additional file 3: **Figure S1.** K-M curve of different age groups. (DOCX 31 kb)

## Abbreviations

ANA: antinuclear antibody
ANCA: anti-neutrophil cytoplasmic antibody
eGFR: estimated glomerular filtration rate
ESRD: End stage renal disease
HSP: Henoch-Schönlein purpura
HSPN: Henoch-Schönlein purpura nephritis
IgA: Immunoglobin A
IgAV: IgA vasculitis
IgAVN: IgA vasculitis nephritis
ISKDC: International Study of Kidney Disease in Children
MAP: mean arterial pressure
Scr: serum creatinine
