# Supplementary material for: Updated Oxford classification and the international study of kidney disease in children classification: application in predicting outcome of Henoch-Schönlein purpura nephritis
Source: Diagn Pathol. 2019 May 10;14:40. doi: 10.1186/s13000-019-0818-0 (PMC6511170; doi:10.1186/s13000-019-0818-0)
Supplement: Supplementary file 1 — Table S1. Clinical and pathological features of patients in different age group. (DOCX 17 kb) [file 13000_2019_818_MOESM1_ESM.docx]

Table S1. Clinical and pathological features of patients in different age group.

| features | Age | | P value |
| --- | --- | --- | --- |
|  | 14-17 y.o. | >18 y.o. |  |
| Patients | 55 | 220 |  |
| MAP (mmHg) | 90.2±9.3 | 96.2±10.7 | <0.001 |
| Scr (μmol/L) | 54.0 (47.0, 69.0) | 68.0(56.0, 88.0) | <0.001 |
| eGFR(ml/min/1.73m^2^) | 143.5 (129.8, 170.4) | 107.3 (81.4, 129.7) | <0.001 |
| Proteinuria（g/24h） | 1.5 (0.75, 3.48) | 1.09 (0.60, 2.85.) | <0.001 |
| M1, n(%) | 9(16.4) | 32（14.5） | 0.735 |
| E1, n(%) | 15(27.7) | 67（30.5） | 0.645 |
| S1, n(%) | 10(18.2) | 139（63.2） | <0.001 |
| T1/T2, n(%) | 0（0） | 8（3.6） | <0.001 |
| C1, n(%) | 26(47.3) | 115（52.3） | 0.595 |
| C2, n(%) | 6（10.9） | 29（13.2） |  |
