# Supplementary material for: Updated Oxford classification and the international study of kidney disease in children classification: application in predicting outcome of Henoch-Schönlein purpura nephritis
Source: Diagn Pathol. 2019 May 10;14:40. doi: 10.1186/s13000-019-0818-0 (PMC6511170; doi:10.1186/s13000-019-0818-0)
Supplement: Supplementary file 2 — Table S2. Immunofluorescence deposition characteristics. (DOCX 15 kb) [file 13000_2019_818_MOESM2_ESM.docx]

| Immunofluorescence deposition,  n (%) | Renal endpoints reached | | P value |
| --- | --- | --- | --- |
|  | Yes | No |  |
| IgM | 19(63.3) | 172（70.2） | 0.441 |
| IgG | 5(16.7) | 21（8.6） | 0.153 |
| IgA | 30(100.0) | 245（100.0） | - |
| C3 | 25(83.3) | 201（82.0） | 0.861 |
| C4 | 3(10.0) | 8(3.3) | 0.076 |

Table S2. Immunofluorescence deposition characteristics.

The data was expressed as numbers (percentages).
