# Supplementary figures and images for: Updated Oxford classification and the international study of kidney disease in children classification: application in predicting outcome of Henoch-Schönlein purpura nephritis
Source: Diagn Pathol. 2019 May 10;14:40. doi: 10.1186/s13000-019-0818-0 (PMC6511170; doi:10.1186/s13000-019-0818-0)

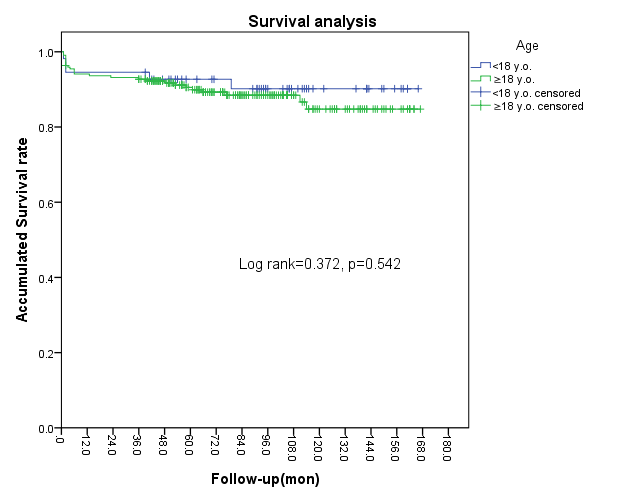


Fig.S1 K-M curve of different age groups.

Supplement: Supplementary file 3 — Figure S1. K-M curve of different age groups. (DOCX 31 kb) [file 13000_2019_818_MOESM3_ESM.docx]
